# Short‐term interleukin‐37 treatment improves vascular endothelial function, endurance exercise capacity, and whole‐body glucose metabolism in old mice

**DOI:** 10.1111/acel.13074

**Published:** 2019-11-21

**Authors:** Dov B. Ballak, Vienna E. Brunt, Zachary J. Sapinsley, Brian P. Ziemba, James J. Richey, Melanie C. Zigler, Lawrence C. Johnson, Rachel A. Gioscia‐Ryan, Rachel Culp‐Hill, Elan Z. Eisenmesser, Angelo D'Alessandro, Charles A. Dinarello, Douglas R. Seals

**Affiliations:** ^1^ Department of Integrative Physiology University of Colorado Boulder Boulder CO USA; ^2^ Department of Medicine University of Colorado Denver Aurora CO USA; ^3^ Department of Internal Medicine Radboud University Medical Center Nijmegen The Netherlands

**Keywords:** aging, AMP‐activated kinase, anti‐inflammatory, oxidative stress

## Abstract

Aging is associated with vascular endothelial dysfunction, reduced exercise tolerance, and impaired whole‐body glucose metabolism. Interleukin‐37 (IL‐37), an anti‐inflammatory cytokine of the interleukin‐1 family, exerts salutary physiological effects in young mice independent of its inflammation‐suppressing properties. Here, we assess the efficacy of IL‐37 treatment for improving physiological function in older age. Old mice (26–28 months) received daily intraperitoneal injections of recombinant human IL‐37 (recIL‐37; 1 µg/200 ml PBS) or vehicle (200 ml PBS) for 10–14 days. Vascular endothelial function (ex vivo carotid artery dilation to increasing doses of acetylcholine, ACh) was enhanced in recIL‐37 vs. vehicle‐treated mice via increased nitric oxide (NO) bioavailability (all *p* < .05); this effect was accompanied by enhanced ACh‐stimulated NO production and reduced levels of reactive oxygen species in endothelial cells cultured with plasma from IL‐37‐treated animals (*p* < .05 vs. vehicle plasma). RecIL‐37 treatment increased endurance exercise capacity by 2.4‐fold, which was accompanied by a 2.9‐fold increase in the phosphorylated AMP‐activated kinase (AMPK) to AMPK ratio (i.e., AMPK activation) in quadriceps muscle. RecIL‐37 treatment also improved whole‐body insulin sensitivity and glucose tolerance (*p* < .05 vs. vehicle). Improvements in physiological function occurred without significant changes in plasma, aortic, and skeletal muscle pro‐inflammatory proteins (under resting conditions), whereas pro‐/anti‐inflammatory IL‐6 was greater in recIL‐37‐treated animals. Plasma metabolomics analysis revealed that recIL‐37 treatment altered metabolites related to pathways involved in NO synthesis (e.g., increased L‐arginine and citrulline/arginine ratio) and fatty acid metabolism (e.g., increased pantothenol and free fatty acids). Our findings provide experimental support for IL‐37 therapy as a novel strategy to improve diverse physiological functions in old age.

## Graphical abstract

Physiological function declines with aging, increasing risk of chronic diseases and disability. We show for the first time that treatment with a novel anti‐inflammatory compound interleukin‐37 (IL‐37) improves multiple physiological functions in old mice, and identify potential roles of reduced superoxide production, improved oxidative metabolism and circulating factors in mediating improvements. Our findings support IL‐37 therapy as a novel strategy for improving diverse physiological functions in old age.

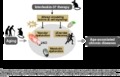

## 1 INTRODUCTION

Older age is characterized by inflammation, oxidative stress, and reduced physiological function, including vascular dysfunction, reduced exercise tolerance, and impaired glucose metabolism. Age‐related vascular dysfunction features impaired endothelial function and stiffening of the large elastic arteries and is a major risk factor for clinical cardiovascular diseases (CVD), cognitive impairments and dementia, chronic kidney diseases, metabolic disorders, and exercise intolerance, among other common conditions of aging (Blacher et al., 2003; Lakatta & Levy, 2003; Waldstein et al., 2008). Reduced exercise tolerance is a sensitive marker of the loss of integrative physiological function with aging and a strong independent predictor of morbidity, disability, and mortality in older adults (Blair et al., 1989; Ekelund et al., 1988). Metabolic dysfunction with aging may manifest as impaired glucose tolerance and/or reduced insulin sensitivity and is responsible for the markedly increased risk of type 2 diabetes mellitus in older adults (Kalyani & Egan, 2013; Selvin, Coresh, & Brancati, 2006). Collectively, these declines in physiological function threaten both the maintenance of healthspan and the achievement of “optimal longevity”—living a long life with extended healthspan (Seals, Justice, & LaRocca, 2016; Seals & Melov, 2014).

Healthy lifestyle interventions, such as regular aerobic exercise and caloric restriction, enhance vascular function, exercise tolerance, and glucose metabolism with aging, in part by reducing inflammation and oxidative stress (Buresh, 2014; Donato et al., 2013; Assar, Angulo, & Rodríguez‐Mañas, 2013; Seals et al., 2016). However, these strategies are limited by several barriers at the population level that restrict consistent adherence (Martens & Seals, 2016; Schutzer & Graves, 2004). As such, there is great interest in novel pharmacological targets and strategies that might enhance physiological function with aging in older adults (Burch et al., 2014; Seals et al., 2016).

One intriguing target for improving physiological function with aging is interleukin‐37 (IL‐37), an anti‐inflammatory cytokine of the interleukin (IL)‐1 family originally characterized in 2010 (Boraschi et al., 2011; Nold et al., 2010). Administration of IL‐37 suppresses inflammation in pro‐inflammatory states and disorders, while reducing morbidity (Ballak et al., 2014; Moretti et al., 2014; Nold et al., 2010; Teng et al., 2014). We recently reported that treatment with recombinant IL‐37 (recIL‐37) improved endurance exercise tolerance in young mice during lipopolysaccharide‐induced inflammation (Cavalli et al., 2017) and improved glucose metabolism in young mice with high‐fat feeding‐induced obesity and inflammation (Ballak et al., 2018). IL‐37 treatment also improved exercise performance in healthy (young) control mice *without* experimentally induced inflammation. Importantly for the present study, these improvements were observed in the absence of reductions in circulating pro‐inflammatory cytokines (Cavalli et al., 2017). Rather, increased exercise capacity in the young animals with IL‐37 administration was related to activation of 5' adenosine monophosphate‐activated protein kinase (AMPK) in the locomotor skeletal muscles (Cavalli et al., 2017). However, the potential efficacy of IL‐37 therapy for improving physiological function in older age has not been investigated.

In the present study, we tested the primary hypothesis that treatment with recIL‐37 would improve vascular function, exercise tolerance, and glucose metabolism in old mice. We also interrogated potential mechanisms of action in vascular tissues and skeletal muscle and conducted a targeted plasma metabolomics analysis to probe possible metabolic pathways involved. Our findings provide the first experimental support for IL‐37 therapy as a novel strategy to improve multiple physiological functions in old age.

## 2 RESULTS

### 2.1 Animal characteristics

Old male C57BL/6N mice (26–28 months) were administered daily intraperitoneal 1 µg injections of recIL‐37 (200 µl of 5 µg/ml in PBS; *N* = 15) or vehicle (200 μl PBS; *N* = 17) for 10–14 days and were sacrificed 24–48 hr following the last injection. RecIL‐37 treatment appeared to be safe and well‐tolerated, as there were no differences in body weight at sacrifice between the groups, nor any differences in mass of key organs (Table 1). Moreover, mortality was not increased with recIL‐37 treatment in these old animals. Indeed, only 3 mice in the IL‐37‐treated group died during the protocol, whereas 6 animals died in the vehicle‐treated group.

**Table 1:** Animal characteristics

	Vehicle	recIL−37
Body mass (g)	32.4 ± 1.7	31.3 ± 1.4
Heart mass (mg)	175 ± 11	169 ± 6
Liver mass (g)	1.69 ± 0.10	2.13 ± 0.33
Pancreas mass (mg)	328 ± 77	300 ± 40
Visceral adipose tissue mass (g)	0.60 ± 0.17	0.41 ± 0.11
Quadriceps muscles mass (mg)	331 ± 12	344 ± 16
Gastrocnemius muscles mass (mg)	288 ± 19	286 ± 7

Note

Data are mean ± *SEM*. *N* = 8–9/group.

### 2.2 Chronic treatment with recIL‐37 improves vascular endothelial function in old mice by increasing NO bioavailability

**Figure 1:**
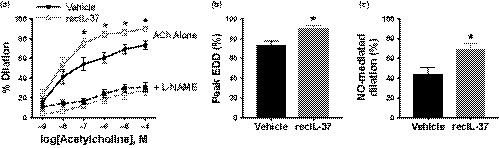
RecIL‐37 treatment improves endothelial function. (a) Endothelium‐dependent dilation (EDD) in isolated carotid arteries to increasing doses of acetylcholine (ACh) alone and in the presence of N^G^‐nitro‐L‐arginine methyl ester (L‐NAME). (b) Peak EDD to acetylcholine. (c) The component of EDD mediated by nitric oxide (NO‐mediated dilation), determined as the difference between peak EDD to ACh alone and EDD in the presence of L‐NAME. Data are mean ± *SEM*. *N* = 9–11 mice per group. *p < .05 vs. vehicle treatment (within time point)

Vascular endothelial function was assessed ex vivo in carotid arteries as EDD to increasing doses of acetylcholine (ACh) (Figure 1). Peak EDD was 74 ± 4% maximal dilation capacity in vehicle‐treated old mice (Figure 1b). RecIL‐37 treatment significantly improved EDD at the four highest doses of ACh (all *p* < .05 vs. vehicle; Figure 1a), as well as peak EDD (*p* = .003 vs. vehicle; Figure 1b).

To assess the role of nitric oxide (NO) in mediating improvements in endothelial function in response to recIL‐37 treatment, dose responses to ACh were repeated in the presence of the NO synthase inhibitor N^G^‐nitro‐L‐arginine methyl ester (L‐NAME), which prevents endothelial NO production. NO‐mediated dilation was calculated as the difference in peak EDD in the absence vs. presence of L‐NAME. In the presence of L‐NAME, group differences in EDD were abolished (dose–response main effect: *p* = .95; Figure 1a). As such, the improvement in vascular endothelial function with recIL‐37 treatment was mediated by an increase in NO bioavailability and NO‐mediated dilation (*p* = .006; Figure 1c).

No differences were observed in endothelium‐independent dilation (EID), as assessed by carotid artery dilation to increasing doses of the NO donor sodium nitroprusside (SNP, 10^–10^ to 10^–4^ M; Supplemental Figure S1), which causes direct (non‐endothelium‐dependent) relaxation of vascular smooth muscle and consequent dilation. The lack of group differences in this response indicates that the observed improvements in ACh‐mediated EDD in the recIL‐37‐treated old mice were not attributable to changes in vascular smooth muscle sensitivity to NO, but rather to events in the vascular endothelium involving increased NO bioavailability.

### 2.3 Enhanced NO bioavailability after recIL‐37 treatment: circulating factors, endothelial cell superoxide production, and oxidant and antioxidant enzyme expression

**Figure 2:**
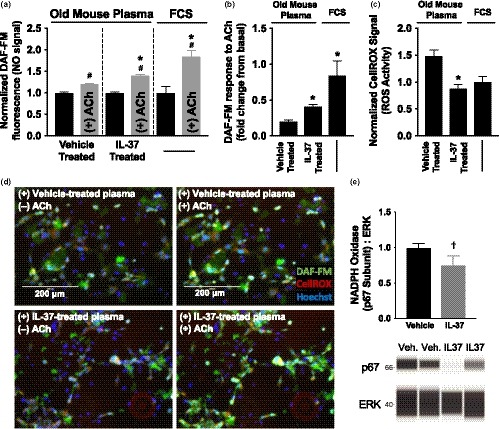
Plasma from recIL‐37‐treated mice increases endothelial cell acetylcholine (ACh)‐stimulated nitric oxide (NO) production and reduces reactive oxygen species production. (a–c) NO production (DAF‐FM fluorescence) before and after incubation with ACh [presented at both time points (a) and as a fold change from basal (b), both normalized within plasma conditions] and reactive oxygen species production (c) (CellROX signal) in human umbilical vein endothelial cells cultured with plasma from vehicle‐ and recIL‐37‐treated old mice or with fetal cow serum (FCS; healthy control condition). Cells were also stained with Hoechst 33342 to indicate healthy nuclei. Data are mean ± *SEM*. *N* = 8–10/group. #*p* < .05 vs. basal conditions (pre‐ACh). *p < .05 vs. vehicle treatment. Each plasma sample was assayed twice over two days. For DAF‐FM experiments, 320 HUVECs were visualized after IL‐37‐treated plasma incubation and 256 cells were visualized after vehicle‐treated plasma incubation. For CellROX experiments, 240 cells were visualized after IL‐37‐treated plasma incubation and 192 cells were visualized after vehicle‐treated plasma incubation. (d) Representative fluorescent images taken at 20X optical zoom. Images are of the same wells incubated with plasma from vehicle‐ (top) or recIL‐37‐treated mice (bottom), before (left) and after (right) the addition of ACh. (e) Protein abundance of NADPH oxidase p67 in aortic lysates with representative Western blotting images generated from electropherograms from the automated capillary electrophoresis system shown below. Data are mean ± *SEM*. *N* = 8–10/group. †*p* < .10 vs. vehicle treatment

To determine the mechanistic role of circulating factors in mediating the effects of recIL‐37 treatment on endothelial function, cultured human umbilical vein endothelial cells (HUVECs) were incubated with 10% plasma from vehicle‐ or recIL‐37‐treated mice for 24 hr under standard conditions. HUVECs were then stained with DAF‐FM diacetate to detect NO release and imaged before and after the addition of ACh to the culture media (see Figure 2d for representative images). ACh‐stimulated NO release was significantly improved in HUVECs incubated with plasma from recIL‐37‐treated vs. vehicle‐treated old mice (*p* < .0001; Figure 2a,b). These results indicate that at least some of the improvements in NO bioavailability and NO‐mediated EDD after recIL‐37 treatment were mediated by changes in circulating factors.

We also stained HUVECs with CellROX to assess endothelial cell reactive oxygen species bioactivity. ROS bioactivity was significantly reduced in HUVECs incubated with plasma from recIL‐37‐treated mice vs. vehicle (*p* < .0001; Figure 2c). These observations indicate that recIL‐37 treatment suppresses endothelial cell ROS bioactivity and that changes in plasma factors contribute to this suppression.

To determine whether recIL‐37 treatment influenced oxidant enzyme expression in mouse vascular tissues, the p67 subunit of the superoxide‐producing enzyme, nicotinamide adenine dinucleotide phosphate oxidase (NADPHO), was measured in aortic lysates using Western immunoblotting. We observed a strong trend toward a reduction in NADPHO p67 with IL‐37 compared with vehicle treatment (*p* = .09; Figure 2e). There was no effect of IL‐37 treatment on aortic expression of the two major isoforms of the superoxide‐clearing antioxidant enzyme, superoxide dismutase (SOD; Supplemental Figure S2, panels B and C). Collectively, these findings suggest that reduced expression of the oxidant enzyme NADPHO, but not increases in SOD, may have contributed to the suppression of vascular ROS production/bioactivity in response to recIL‐37 treatment and, therefore, less scavenging of NO and increases in NO bioavailability.

### 2.4 RecIL‐37 treatment, arterial stiffness, and blood pressure

We next determined if the improvement in vascular endothelial function with recIL‐37 treatment was accompanied by reductions in large elastic artery stiffness and/or systolic blood pressure, both of which increase with aging and accompany endothelial dysfunction (Lakatta, 2003; Nigam, Mitchell, Lambert, & Tardif, 2003). Arterial stiffness was assessed noninvasively in vivo using the gold standard measure of aortic pulse wave velocity (PWV) before and after treatment with recIL‐37 or vehicle (Table 2). Contrary to our hypothesis, aortic PWV was nonsignificantly but slightly *increased* in both groups across the intervention (main effect of time: *p* = .32), possibly due to stress associated with the daily IP injections. However, there was no independent effect of recIL‐37 treatment on aortic PWV (group x time interaction: *p* = .91).

**Table 2:** Pulse wave velocity and blood pressure

	Vehicle		recIL‐37	
	Pre	Post	Pre	Post
Aortic pulse wave velocity (cm/s)	364 ± 41	397 ± 29	362 ± 30	404 ± 28
Blood pressure (mmHg)				
Systolic	97 ± 2	99 ± 2	98 ± 2	95 ± 3
Diastolic	68 ± 1	67 ± 1	69 ± 2	65 ± 2

Note

Data are mean ± *SEM*. *N* = 8–10/group.

Blood pressure also was assessed noninvasively in vivo using the tail cuff method before and after recIL‐37 or vehicle treatment (Table 2). At baseline, systolic blood pressure averaged 98 mmHg, which is consistent with what we and others have previously observed in older mice of this strain (C57BL/6N) (Gioscia‐Ryan et al., 2018; Toth et al., 2013)—although tail cuff blood pressure is typically lower than blood pressure measured elsewhere in the body, it is still sensitive enough to detect changes across interventions and/or across groups. In the present study, there were no differences in systolic blood pressure before and after recIL‐37 or vehicle treatment (main effect of time: *p* = .89; time x group interaction: *p* = .80). There was a modest, but statistically significant decrease in diastolic blood pressure across the treatment period (main effect of time: *p* = .02), but no independent effect of the recIL‐37 treatment (time x group interaction: *p* = .57). These findings indicate that the improvements in vascular endothelial function with recIL‐37 treatment in old mice are not secondary to reductions in arterial stiffness and/or systolic blood pressure.

### 2.5 Treatment with recIL‐37 improves endurance exercise capacity and induces AMPK activation in locomotor skeletal muscle in old mice

**Figure 3:**
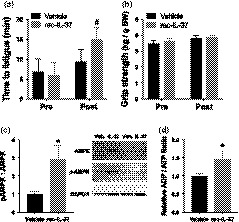
RecIL‐37 treatment improves skeletal muscle function. (a) Time to fatigue on the endurance rota‐rod test and (b) normalized grip strength, measured pre‐ and post‐vehicle or recIL‐37 treatment. *N* = 10–13 mice per group. (c) Relative ratio of phosphorylated‐AMPK (p‐172) and total AMPK in quadriceps lysate from vehicle‐ and recIL‐37‐treated mice, with representative Western blot images shown to the right. Western blot analyses were performed in duplicate in tissue lysates from 10–12 mice per group. (d) ADP/ATP ratio in collagenase‐separated quadriceps muscle cells from vehicle‐ or recIL‐37‐treated mice. Enzymatic assays were performed in duplicated for each muscle tissue sample from 10 to 12 mice per group. All data are mean ± *SEM*. *p < .05 vs. vehicle. #*p* < .05 vs. pre‐intervention

Motor function was assessed before and after the intervention period as time to fatigue on an endurance rota‐rod test and grip strength. We observed a 2.4‐fold increase in endurance capacity following recIL‐37 treatment (*p* < .01 vs. pre‐intervention; Figure 3a), but no effects on grip strength (main effect: *p* = .68; Figure 3b). Vehicle treatment did not affect endurance capacity or grip strength (*p* > .56).

To investigate potential mechanisms for improved endurance exercise capacity with recIL‐37 treatment, we measured the ratio of phosphorylated to total AMPK (i.e., AMPK activation), a key regulator of cellular energy homeostasis and oxidative metabolism, as well as the ratio of ADP/ATP, an indicator of increased oxidative metabolism and key signal for AMPK activation, in quadriceps skeletal muscle. Both AMPK activation (+2.9‐fold) and the ADP/ATP ratio (+50%) were significantly increased after recIL‐37 treatment (both *p* < .05; Figure 3c,d). Importantly, the activation of AMPK by recIL‐37 treatment appears to be specific to skeletal muscle, as AMPK activation was not greater in aortic lysates with recIL‐37 vs. vehicle treatment (aortic pAMPK:AMPK ratio: vehicle 1.00 ± 0.23 A.U. vs. recIL‐37 0.76 ± 0.09 A.U., *p* = .29). These changes were independent of effects on oxidative stress, as quadricep muscle abundance of nitrotyrosine, SOD1, and SOD2 was not different in the recIL‐37‐ vs. vehicle‐treated animals (*p* = .33–.98, Supplemental Figure S2, panels D–F).

Consistent with our previous findings (Cavalli et al., 2017), these results demonstrate that recIL‐37 treatment dramatically improves endurance exercise capacity (exercise tolerance) and this is accompanied by a marked increase in AMPK activation in locomotor skeletal muscle of old mice.

### 2.6 Treatment with recIL‐37 improves whole‐body glucose metabolism in old mice

**Figure 4:**
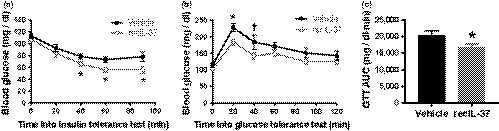
RecIL‐37 treatment improves insulin sensitivity and glucose tolerance. (a) Blood glucose concentrations over 90 min following intraperitoneal injection of insulin (0.75 U/kg body weight). *N* = 12–14 mice per group. (b) Blood glucose concentrations over 120 min following intraperitoneal injection of glucose (2 mg/g body weight). *N* = 8–10 mice per group. (c) Area under the curve blood glucose concentration over the 120‐min glucose tolerance test. Data are mean ± *SEM*. *p < .05 vs. vehicle treatment (within time point). †*p* < .10 vs. vehicle treatment (within time point)

Insulin sensitivity, as assessed by the reduction in blood glucose concentrations following IP insulin injection (insulin tolerance test), was significantly improved by recIL‐37 treatment (main effect of group × time: *p* < .05 Figure 4a). In addition, blood glucose concentrations in response to IP glucose injection (glucose tolerance test) were attenuated by recIL‐37 treatment, particularly over the initial 60 min post‐glucose challenge (Figure 4b), as was the area under the curve for the blood glucose response across the 2 hr following glucose injection (Figure 4c). Basal insulin concentrations in the pancreas were not different in the two groups (Supplemental Figure S3). Taken together, these observations suggest that treatment with recIL‐37 modestly improves whole‐body insulin sensitivity and glucose tolerance in old mice, without effects on basal pancreatic insulin concentrations.

### 2.7 RecIL‐37 treatment on inflammatory cytokines and chemokines

We measured inflammatory protein markers in plasma and in both aorta and skeletal muscle. Data are summarized in Supplemental Figure S4. Consistent with our previous work, concentrations of IL‐6 in plasma (*p* = .09), aorta lysates (*p* < .05), and quadriceps lysates (*p* = .10) were or tended to be higher in recIL‐37‐ vs. vehicle‐treated mice (Cavalli et al., 2017), and concentrations of anti‐inflammatory IL‐1 receptor antagonist (IL‐1Ra) tended to be higher in plasma (*p* = .09) in recIL‐37‐ vs. vehicle‐treated mice (Ballak et al., 2018). Concentrations of KC (murine chemokine ligand 1, CXCL1) were higher in plasma of recIL‐37 animals (*p* < .05). We observed no significant differences in any other inflammatory markers in plasma, aorta, and/or skeletal muscle, including TNFα, IFNγ, and IL‐1β, in recIL‐37‐ vs. vehicle‐treated animals. Mean aortic abundance of total nuclear factor‐kappa B (NF‐κB) and phosphorylated NF‐κB were not significantly different in recIL‐37‐treated and control mice (*p* ≥ .14), and the ratio of phosphorylated to total NFκB was similar in the two groups (*p* = .94; Supplemental Figure S5). In summary, although we observed increases in select cytokines with recIL‐37 treatment, there were no changes in the majority of markers assessed and thus no consistent anti‐inflammatory effect of this treatment in older mice. As such, the improvements in physiological function with recIL‐37 treatment described above were not obviously mediated by robust systemic reductions in inflammation, but rather by other signaling mechanisms.

### 2.8 Changes in circulating metabolites with recIL‐37 treatment: plasma metabolomics

**Figure 5:**
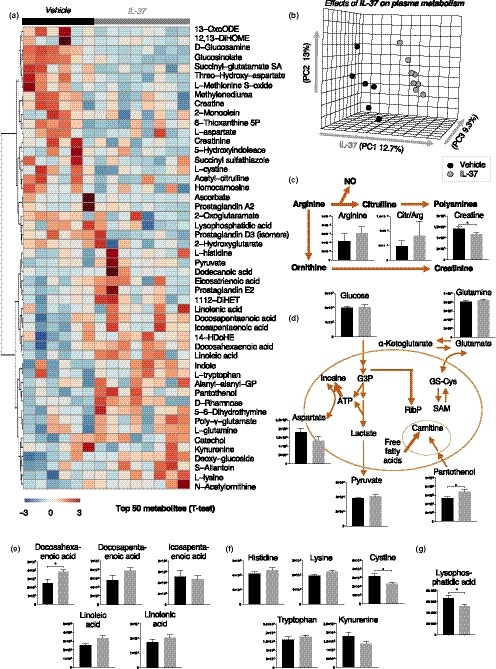
Treatment with recIL‐37 affects levels of certain circulating metabolites. (a) Heat map of key metabolites in plasma. (b) Principal component analysis based on partial least squares‐discriminant analysis. Plasma levels of (c) L‐arginine and related metabolites, (d) pantothenol (bottom right of panel) and related metabolites, (e) key free fatty acids, including docosahexaenoic acid (DHA, first graph in panel), (f) select amino acids and their metabolites and (g) lysophosphatidic acid. Data are mean ± *SEM* of the integrated peak areas (i.e., area under the curve) from ion chromatograms for each metabolite. *N* = 6–8 mice per group. *p < .05 across groups (Student's unpaired *t* test)

The results of our ex vivo plasma exposure experiments in HUVECs suggest that improved NO‐mediated vascular endothelial function with recIL‐37 treatment may by mediated by alteration of circulating factors. To explore the possible identity of such factors, metabolomics analyses of mouse plasma were performed, including detection of common metabolites, catabolites, and lipid markers. A heat map of the top 50 metabolic changes (sorted by unpaired *t* test) between vehicle‐treated and recIL‐37‐treated mice is shown in Figure 5a. Group medians, fold changes, and p‐values are extensively reported in tabulated form in Supplemental Table S1. Principal component analysis based on partial least squares‐discriminant analysis demonstrated distinct clustering of samples from vehicle and recIL‐37‐treated mice (Figure 5b), with principal component 1 (PC1) explaining ~13% of the total variance across sample; variable importance in the projection [VIP] scores for the PLS‐DA model is provided in Supplemental Figure S6.

Levels of several metabolites related to arginine (the precursor for NO) metabolism were or tended to be different across groups (see Table S1), overall suggesting a shift toward greater NO production. In particular, creatine, a byproduct of conversion of arginine to ornithine, was significantly lower in recIL‐37‐treated mice (*p* < .05), accompanied by slight, albeit nonsignificant, increases in L‐arginine and the ratio of L‐citrulline to L‐arginine (an indicator of increased NO synthesis) (Figure 5c). RecIL‐37‐treated mice also had significantly higher circulating levels of pantothenol (*p* < .05), a precursor of coenzyme A (Figure 5d, bottom right of panel), higher levels of free fatty acids, including cardioprotective docosahexaenoic acid (DHA; *p* < .05) (Figure 5e), and lower levels of cystine (*p* < .05) and kynurenine (*p* = .15) (Figure 5f), which suggest a decrease in oxidative stress and inflammation. Lastly, recIL‐37 treatment decreased circulating levels of cardiotoxic lysophosphatidic acid (Figure 5g, *p* < .05). There were no differences in levels of any other amino acids (other than L‐arginine) or metabolites related to energy homeostasis that have been reported to change with recIL‐37 treatment in young mice (Cavalli et al., 2017) (Supplemental Figure S7), indicating different mechanisms may be involved in recIL‐37‐mediated improvements in endurance in old vs. young mice. In summary, recIL‐37 treatment in old mice altered the circulating concentrations of selective metabolites linked to NO production and endothelial function, as well as fatty acid metabolism/skeletal muscle function.

## 3 DISCUSSION

Here, we investigated for the first time the potential efficacy of treatment with exogenous IL‐37 as a “later life” intervention for improving multiple physiological functions with aging. To do so, 26–28 month‐old mice, equivalent in age to 80‐ to 85‐year‐old humans, were treated for 10–14 days with intraperitoneal injections of recIL‐37 or vehicle. Overall, our results demonstrate that treatment with recIL‐37 improves multiple clinically relevant domains of physiological performance in old mice, including vascular endothelial function, endurance exercise capacity, and whole‐body glucose metabolism. As such, our findings provide critical proof‐of‐concept experimental evidence for further translation of IL‐37 as a potential therapeutic compound for extending healthspan.

### 3.1 IL‐37 signaling and actions

IL‐37 was identified in 2010 as an anti‐inflammatory agent with diverse effects on cellular signaling (Boraschi et al., 2011; Nold et al., 2010). Upon injection in vivo, recIL‐37 rapidly binds to its receptors IL‐18Rα and IL‐1R8 (formerly TIR8 or SIGIRR) to initiate downstream cellular signaling and is then rapidly cleared from the blood (Cavalli & Dinarello, 2018). Depending on the physiological state, these events may influence pro‐inflammatory signaling via inhibition of nuclear factor‐kappa B (NF‐κB) and mitogen‐activated protein kinase (MAPK) intracellularly (Cavalli & Dinarello, 2018). However, IL‐37 has other cellular and systemic effects, including activation of 5' adenosine monophosphate‐activated protein kinase (AMPK), which in turn reduces mTOR signaling (Ballak et al., 2014; Cavalli et al., 2017; Nold‐Petry et al., 2015). Although IL‐37 is not produced endogenously in mice, its receptors are present and crucial for mediating effects of IL‐37 (Nold‐Petry et al., 2015), thereby mimicking human IL‐37 signaling. As such, despite lack of endogenous production, administration of recIL‐37 to mice may provide insight into the effects of this molecule on physiological function in humans.

### 3.2 Cardiovascular function

A primary physiological function of interest in this study was vascular endothelial function, as assessed by ACh‐induced EDD of isolated carotid arteries. We found that recIL‐37 improved endothelial function by increasing NO bioavailability. Impaired NO‐mediated EDD is a major antecedent to numerous chronic clinical disorders of aging, including atherosclerotic diseases (e.g., coronary artery disease), hypertension, cognitive dysfunction, chronic kidney disease, and exercise intolerance (Gimbrone & García‐Cardeña, 2016; Johnson et al., 2017; Van Craenenbroeck et al., 2016). As such, improvements in endothelial function with IL‐37 may lead to novel therapies to reduce the risk of these age‐related medical conditions in humans.

The improvements in vascular endothelial function with IL‐37 treatment were independent of changes in vascular smooth muscle sensitivity to NO, arterial stiffness, or arterial blood pressure. Rather, the results of our mechanistic ex vivo experiments in HUVECs incubated with plasma from recIL‐37 vs. vehicle‐treated animals indicate that the enhanced NO bioavailability and EDD after IL‐37 treatment were likely due to greater NO production and reduced ROS synthesis, which, in endothelial cells, is predominantly the NO scavenger superoxide. The recIL‐37 treatment‐induced reduction in endothelial cell ROS production was, in turn, accompanied by reduced expression of the superoxide‐generating enzyme NADPHO, but without any compensatory increase in the major superoxide‐scavenging antioxidant enzyme SOD (Donato et al., 2007; van der Loo et al., 2000). Collectively, our findings suggest that IL‐37‐mediated improvements in NO bioavailability and endothelial function may have occurred through reduced NADPHO‐associated increases in superoxide production and oxidative stress, although further investigation is needed to confirm these mechanisms in vivo.

The results of these ex vivo plasma exposure experiments in HUVECs also suggest an important role of circulating factors in transducing at least some of the benefits of IL‐37 treatment on vascular endothelial function. Importantly, as IL‐37 is rapidly cleared from the blood following injection (Cavalli & Dinarello, 2018), there should have been minimal to no IL‐37 remaining in the plasma at the time of sacrifice (when blood was collected for these cell culture experiments). To confirm this, we measured plasma concentrations of IL‐37 using a commercially available ELISA kit (AdipoGen) and found no detectable IL‐37 in the plasma from either group. It is important to note that the kits that are currently available are nonspecific, also reacting with other cytokines within the IL‐1 family (primarily IL‐1Ra); as such, they are *not* appropriate for quantifying nonzero IL‐37 concentrations. However, here, we can safely conclude that there was no IL‐37 in the plasma that was used for cell culture experiments. As such, effects must be due to other circulating factors altered by IL‐37 treatment rather than due to direct effects of IL‐37.

As noted previously, IL‐37 treatment had no effect on arterial stiffness or blood pressure. NO is an important regulator of both of these functions. However, although we observed improved NO bioavailability under stimulated conditions (ACh‐induced) in the recIL‐37‐treated animals, there were no differences in *tonic* NO production, which would be necessary to affect resting arterial stiffness and blood pressure. Additionally, the short duration of the intervention likely was insufficient to induce changes in arterial wall structure necessary to reduce intrinsic aortic stiffness. Indeed, previous intervention studies in our laboratory that have observed changes in the structural component of arterial stiffness have used longer treatment durations than in the present investigation (e.g., Gioscia‐Ryan et al., 2018; Sindler et al., 2011). Thus, it is possible that longer‐term treatment with IL‐37 may have de‐stiffening and/or blood pressure‐lowering effects.

### 3.3 Endurance exercise capacity

Endurance exercise capacity is a well‐established measure of integrative physiological function and one of the strongest independent predictors of morbidity and mortality in humans (Blair et al., 1989; Ekelund et al., 1988). Consistent with our previous observations in young mice (Cavalli et al., 2017), we found that chronic treatment with recIL‐37 induced an ~2.5‐fold increase in endurance in our old mice. As such, our findings provide preclinical evidence for exploring IL‐37 as a potential therapy for improving exercise capacity with aging.

Biochemical assessment of quadriceps muscle from our mice, a locomotor skeletal muscle that is critically involved in determining exercise performance, revealed an ~3‐fold greater ratio of phosphorylated to total AMPK, as well as a 50% increase in the ADP‐ATP ratio, in mice treated with recIL‐37, suggesting that IL‐37 improves skeletal muscle endurance via enhancing oxidative metabolism. These observations add to our previous findings that IL‐37 activates AMPK in immune cells, adipocytes, and skeletal muscle from young mice (Ballak et al., 2014; Nold et al., 2010; Nold‐Petry et al., 2015).

### 3.4 Whole‐body glucose metabolism

We observed improved whole‐body glucose metabolism with IL‐37 treatment in old mice, as assessed by glucose and insulin tolerance tests. These observations support the possibility that IL‐37 treatment may be effective for enhancing whole‐body glucose tolerance and insulin sensitivity with aging. Unfortunately, the comprehensive multi‐domain physiological phenotyping performed in the present study restricted our ability to properly investigate the signaling pathways involved under the required conditions of using glucose‐ or insulin‐stimulated tissues. We did measure basal pancreatic insulin concentrations and found that they were similar in the two groups of animals; however, that does not rule out an effect of recIL‐37 on insulin secretion during the metabolic tests. As such, the intracellular mechanisms underlying the effects of IL‐37 on glucose metabolism in old mice will need to be elucidated in a follow‐up investigation designed specifically to investigate this functional improvement.

### 3.5 Markers of inflammation

In agreement with our earlier work on recIL‐37 administration in young adult mice (Cavalli et al., 2017), IL‐37 treatment did not uniformly influence concentrations of pro‐inflammatory proteins. Specifically, plasma, aorta, or skeletal muscle concentrations of TNFα, IFNγ, and IL‐1β were not significantly different in the old mice treated with recIL‐37 compared with vehicle.

Also consistent with previous findings in young mice (Cavalli et al., 2017), concentrations of IL‐6 generally were greater in the recIL‐37‐ vs. vehicle‐treated old mice in the present study. IL‐6 can function as both pro‐ and anti‐inflammatory molecule, depending on its tissue origin of synthesis and release, the cell type it is acting on, and the context (Hunter & Jones, 2015). Although chronically elevated levels of IL‐6 are involved in the pathogenesis of several diseases (Unver & McAllister 2018), acute increases in IL‐6 can have *anti*‐inflammatory effects in some physiological settings, for example, as part of controlling innate immune system responses (Hegde, Pahne, & Smola‐Hess, 2004; Hunter & Jones, 2015) and when released from skeletal muscle in response to exercise (Pedersen & Febbraio, 2008). As such, it is possible that the increases in plasma and tissue IL‐6 concentrations with recIL‐37 treatment reflect an overall anti‐inflammatory action, particularly in combination with increases in anti‐inflammatory IL‐1Ra. IL‐6 also can have direct signaling effects. In skeletal muscle, IL‐6 has been linked to improved endurance capacity via regulation of AMPK (Kelly et al., 2004), muscle hypertrophy (Serrano, Baeza‐Raja, Perdiguero, Jardí, & Muñoz‐Cánoves, 2008), and skeletal muscle fat oxidation (Fäldt et al., 2004; Wolsk, Mygind, Grøndahl, Pedersen, & Hall, 2010). Moreover, increased circulating IL‐6 levels can also stimulate greater release of glucagon‐like peptide‐1 from the pancreas, which in turn can stimulate greater insulin release and enhance glucose tolerance (Ellingsgaard et al., 2011). Although we found no differences in basal insulin concentrations in the pancreas, it is still possible recIL‐37 treatment may have improved glucose‐stimulated pancreatic insulin *secretion*. In any case, the potential effects of IL‐6 on increased endurance capacity and improved glucose tolerance with IL‐37 treatment merit further investigation in the future.

Lastly, plasma concentrations of KC were greater in our IL‐37‐treated mice compared with control mice. Although generally viewed as pro‐inflammatory, KC also can act in an anti‐inflammatory manner under certain conditions (Bachmaier, Toya, & Malik, 2014). Thus, as with IL‐6, the pro‐ vs. anti‐inflammatory effects of increases in circulating KC on physiological improvements with IL‐37 administration remain to be elucidated.

### 3.6 Plasma metabolomics analysis

We performed a targeted metabolomics analysis of plasma as an initial step in identifying the circulating factors by which recIL‐37 treatment improved vascular endothelial function. Among the metabolites differing in recIL‐37 vs. vehicle‐treated animals, several were linked to enhanced NO signaling, and reduced oxidative stress and inflammation. Of particular interest were differences in metabolites related to L‐arginine metabolism. Creatinine, a downstream byproduct of conversion of arginine to ornithine, was lower in recIL‐37‐treated mice, indicating a shift in arginine metabolism toward less arginine metabolized to ornithine and more available for NO synthase conversion to NO and citrulline. The ratio of L‐arginine to L‐citrulline tended to be higher in plasma from recIL‐37 vs. placebo‐treated mice, which also is consistent with greater NO synthesis. Moreover, we observed changes in metabolites that may reflect reduced inflammation and oxidative stress, including lower levels of cystine and kynurenine (González Esquivel et al., 2017) and higher levels of the cardioprotective free fatty acid DHA (Innes & Calder, 2018; Sakai et al., 2017). Indeed, supplementation with DHA, alone and in combination with eicosapentaenoic acid, improves NO‐mediated endothelial function in humans with CVD risk factors (Goodfellow, Bellamy, Ramsey, Jones, & Lewis, 2000; Mori et al., 2000; Rizza et al., 2009). In the absence of metabolic tracing, increased levels of DHA and other free fatty acids are also suggestive of increased lipid mobilization and catabolism, both of which may have contributed to improved skeletal muscle function. Along these lines, we additionally observed that recIL‐37‐treated mice had greater concentrations of the coenzyme A precursor pantothenol. Coenzyme A is required for mitochondrial metabolism, improves fatty acid metabolism, and is linked to AMPK phosphorylation (Fediuc, Gaidhu, & Ceddia, 2006), thus providing another potential mechanism by which recIL‐37 treatment may improve skeletal muscle endurance. Overall, these observations suggest that plasma metabolomics analyses may be helpful in identifying molecular signatures of the mechanistic processes through which novel treatments such as recIL‐37 may exert their beneficial physiological effects with aging.

### 3.7 Conclusions and perspectives

In conclusion, the results of this initial study of the effects of IL‐37 treatment in the setting of physiological aging indicate that recIL‐37 improves vascular endothelial function, endurance exercise capacity, and whole‐body glucose metabolism in old mice. Our findings also provide insight into the potential mechanisms mediating the beneficial effects of IL‐37 therapy on vascular function and locomotor skeletal muscle endurance.

To date, several studies have investigated the potential role of natural variants of the IL‐37 gene in human disease, including *M. tuberculosis* infection (Liu et al., 2017), coronary artery disease (Yin et al., 2017), and rheumatoid arthritis (Pei et al., 2013). In general, variants linked to increased production of IL‐37 and/or greater IL‐37 signaling are associated with lower disease burden. However, more research is needed regarding the identification of IL‐37 gene variants that influence IL‐37 protein abundance/signaling and the physiological impact of those variants. The present findings provide essential preclinical evidence for recIL‐37 as a potential treatment for age‐related impairments in physiological function, physical disability, and chronic cardio‐metabolic disease risk.

## 4 EXPERIMENTAL PROCEDURES

Detailed descriptions of all experimental procedures are provided in the supplement, as well as statistical analyses. All experiments were approved by the Institutional Animal Care and Use Committee at the University of Colorado Boulder (protocol #2539) and conformed to the standards set forth by the National Institutes of Health Guide for the Care and Use of Laboratory Animals.

Old male C57BL/6N mice were obtained from the National Institute on Aging colony maintained by Charles River at 20–24 months of age. Male mice of this strain and species demonstrate many clinical aspects of human aging, including vascular endothelial dysfunction (Rippe et al., 2010; Sindler et al., 2011), whereas female mice do not as their hormone profile is not consistent with female older adult humans. All mice were multi‐housed in a conventional facility on a 12‐hr light/dark cycle and given ad libitum access to standard rodent chow and normal drinking water. Following experiments, mice were euthanized prior to terminal measures by exsanguination via cardiac puncture while maintained under inhaled isoflurane anesthesia.

Following in vivo baseline testing, old mice received daily IP injections of 1 μg recIL‐37 (*N* = 15) in 200 μl PBS or vehicle (200 μl PBS; *N* = 17) for 10–14 days (Ballak et al., 2018). The recombinant human IL‐37 that was used in these experiments was expressed in *E. coli* with an N terminus at valine 46 and C terminus at residues 218 and purified to homogeneity, as reported previously (Li et al., 2015). In vivo post‐testing was initiated following at least 6 days of injections (up to 6 consecutive days of testing), with daily injections continued throughout post‐testing. In vivo testing included assessment of aortic PWV, tail cuff blood pressure, grip strength, endurance on a rota‐rod, and glucose and insulin tolerance tests (only post‐intervention), as described in the supplement.

Mice were euthanized 24–48 hr following the last injection in order to ensure stress associated with the IP injections did not affect vascular endothelial function measures. Immediately following sacrifice, the carotid arteries were excised and cannulated onto glass pipette tips in pressure myograph chambers (DMT, Inc.) for assessment of EDD and EID, as described in detail in the supplement. Plasma was collected for experiments in which cultured human umbilical vein endothelial cells (HUVECs; Lonza) were exposed to plasma from vehicle‐ vs. recIL‐37‐treated mice to assess NO bioavailability and ROS production, and for metabolomics analysis. The aorta and quadriceps muscles were collected and flash frozen for later analysis of protein abundance (Western blotting), skeletal muscle ADP:ATP ratio, and concentrations of inflammatory cytokines and chemokines. Please see the supplement for detailed descriptions of all analyses.

## AUTHOR CONTRIBUTIONS

D.B.B., L.C.J., R.A.G.‐R., C.A.D., and D.R.S. conceived the experiments. D.B.B., V.E.B., B.P.Z., M.C.Z., L.C.J., R.A.G.‐R., A.D'A., and D.R.S. designed the experiments. E.Z.E. developed the protocol for purifying human recIL‐37 and provided it for these experiments. D.B.B., V.E.B., Z.J.S., B.P.Z., J.J.R, M.C.Z., L.C.J., and R.C‐H. collected the data. V.E.B., Z.J.S., B.P.Z., R.C‐H., and A.D'A. analyzed the data. D.B.B., V.E.B., B.P.Z., A.D'A., C.A.D., and D.R.S. interpreted the data. V.E.B. and D.R.S. drafted the manuscript. All authors revised the manuscript critically for intellectual content. All authors approved the final version of the manuscript.

## Data Availability

All data are available from the corresponding author upon reasonable request.
