# Supplementary material for: Short‐term interleukin‐37 treatment improves vascular endothelial function, endurance exercise capacity, and whole‐body glucose metabolism in old mice
Source: Aging Cell. 2019 Nov 21;19(1):e13074. doi: 10.1111/acel.13074 (PMC6974720; doi:10.1111/acel.13074)
Supplement: Supplementary file 1 [file ACEL-19-e13074-s001.docx]

**SUPPLEMENTAL MATERIAL TO:**

**Short-term interleukin-37 treatment improves vascular endothelial function, endurance exercise capacity and whole-body glucose metabolism in old mice**

**Dov B. Ballak**^1,2*^ **& Vienna E. Brunt**^1*^**, Zachary J. Sapinsley**^1^**, Brian Ziemba**^1^**, James J. Richey**^1^**, Melanie C. Zigler**^1^**, Lawrence C. Johnson**^1^**, Rachel A. Gioscia-Ryan**^1^**, Rachel Culp-Hill**^2^**, Elan Z. Eisenmesser**^2^**, Angelo D’Alessandro**^2^**, Charles A. Dinarello**^2,3^**, Douglas R. Seals**^1^

*DBB and VEB share first authorship

^1^ Department of Integrative Physiology, University of Colorado Boulder, Boulder, Colorado, USA

^2^ Department of Medicine, Anschutz Medical Campus, University of Colorado Denver, Aurora, Colorado, USA

^3^ Department of Internal Medicine, Radboud University Medical Center, Nijmegen, The

Netherlands

**Supplemental Figures**

**Supplemental Figure S1.** Endothelium-independent dilation in isolated carotid arteries to increasing doses of sodium nitroprusside. Data are mean ± S.E.M. N=8-11 mice per group.

**Supplemental Figure S2. Tissue markers of oxidative stress in response to IL-37 treatment.** Nitrotyrosine, the cytosolic isoform of superoxide dismutase [SOD]1, and the mitochondrial isoform of SOD [SOD2]) in thoracic aorta lysates (A-C) and quadriceps muscle lysates (D-F). Representative Western blot images are shown below. Data are mean ± SEM. N=10-12 per group.

**Supplemental Figure S3. No effect of recIL-37 treatment on basal pancreatic insulin content.** Measured in pancreas lysates in duplicate by ELISA. N=6 mice per group. Data are mean ± SEM.

**Supplemental Figure S4.** **Plasma and tissue cytokine levels in response to IL-37 treatment.** Plasma concentrations of interleukin (IL)-6 (A), IL-1 receptor antagonist (IL-1ra) (B) and KC (CXCL1) (c). IL-6 (D), Tumor necrosis factor (TNF)α (E), interferon (IFN)γ (F), and IL-1β (G) in quadriceps muscle and thoracic aorta lysates. Measured in duplicate by ELISA. N=10-12 mice per group. Data are mean ± SEM. *p<0.05 vs. vehicle. †p<0.10 vs. vehicle.

**Supplemental Figure S5.** Abundance of phosphorylated **(A)**, total **(B)**, and the ratio of phosphorylated to total **(C)** nuclear factor-kappa B (NFκB) in thoracic aorta lysates. **D)** Representative Western blot images. Data are mean ± SEM. N=10-12 per group.

**Supplemental Figure S6.** Variable Importance in the Projection (VIP) scores for the partial least squares-discriminant analysis (PLS-DA) model provided in Figure 6A. From targeted metabolomics analysis of plasma from vehicle and recIL-37-treated mice. The VIP score is a weighted sum of squares of the PLS loadings that takes into account the amount of explained Y-variation in each direction. **
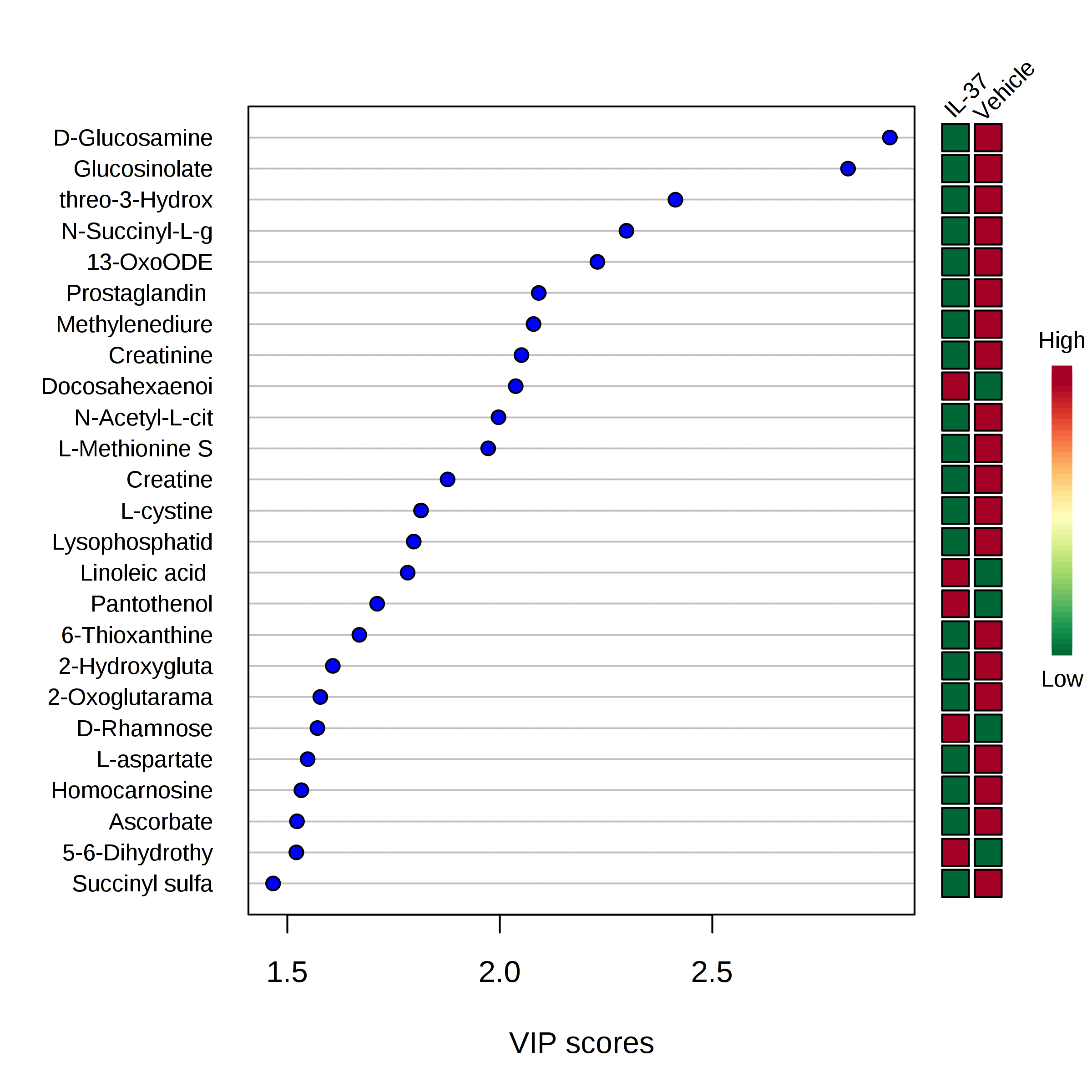
**

**Supplemental Table S1.** Complete results of the targeted metabolomics analysis of plasma from vehicle and recIL-37-treated mice.

**Supplemental Figure S7**. Treatment with recombinant IL-37 did not affect circulating levels of metabolites associated with amino acid metabolism or energy homeostasis. Data are mean ± S.E.M. of the integrated peak areas (i.e., area under the curve) from ion chromatograms for each metabolite.

**Experimental Procedures**

***Vascular Endothelial Function***

Immediately following sacrifice, the carotid arteries were excised, cannulated onto glass pipette tips in pressure myograph chambers (DMT, Inc.; Aarhus, Demark) containing EDTA-buffered physiological saline solution, pressurized to 50 mmHg and allowed to equilibrate for 45 min prior to assessment of EDD. Following preconstriction with phenylephrine (2 μM; Sigma-Aldrich Corp., St. Louis, MO), EDD was assessed by measuring the increase in luminal diameter in response to increasing concentrations of ACh (1x10^-9^ to 1 x 10^-4^ M; Sigma-Aldrich Corp.), first in the absence and then in the presence of 0.1 mM L-NAME (30 min pre-incubation; Sigma-Aldrich Corp.). NO-mediated dilation was calculated as the difference between peak dilation to ACh alone and in the presence of L-NAME. EID was assessed as dilation in response to increasing doses of SNP (1x10^-10^ to 1 x 10^-4^ M; Sigma-Aldrich Corp.). To account for baseline differences in vessel diameter, all dose response data are reported as a percentage of maximal dilation (9).

***Plasma Exposure NO bioavailability and ROS production Cell Culture Experiments***

Human umbilical vein endothelial cells (HUVECs; Lonza, Basel, Switzerland) were cultured and passaged (10 passages maximum) at 37°C and 5% CO_2_ to ~80% confluency in EGM-2 media (Lonza) supplemented with 10% fetal calf serum (FCS; Sigma-Aldrich Corp.), 100 U/mL penicillin and 100 µg/mL streptomycin (Gibco, Gaithersburg, MD). Cells were washed with Hanks buffered salt solution (HBSS), lifted with TrypLE cell dissociation enzyme (Gibco), washed and resuspended in HBSS, counted and diluted to 9 x 103 cells/mL (designed to result in 60-80% confluency over 48-hour incubation) in FCS-supplemented EGM-2. 100 µL of HUVEC cell suspension was added to each well of a high content imaging plate with 0.2 mm glass bottom (Corning, Kennebunk, ME) coated with bovine gelatin substrate (Attachment Factor Solution; Cell Applications, Inc., San Diego, CA) to facilitate cell adhesion. After a 24-hour incubation, attached cells were washed once with Dulbecco’s phosphate-buffered saline (D-PBS) and incubated in FCS-free EGM-2 media supplemented with 10% mouse plasma for 24 hours (37°C, 5% CO_2_). Attached cells were then washed twice with HBSS and incubated for 60 minutes (37°C, 5% CO_2_) with a fluorescent probe mixture [1.8 µM DAF-FM diacetate nitric oxide probe, 1.8 µg/mL Hoechst 33342 nuclear probe, 9.2 µM CellROX Deep Red superoxide probe and 0.04% Pluronic F127 (all from Invitrogen, Carlsbad, CA) in HBSS]. After dye removal and three washes with HBSS, cells were left in 100 µL of HBSS for subsequent imaging. Microscopy was carried out on an EVOS FL microscope (Invitrogen, Carlsbad, CA) set at 20X magnification. Cells were selected for imaging based on characteristic endothelial cell and nuclear morphology. Fluorescent imaging of cytoplasmic DAF-FM before and 5 min after addition of 200 µM ACh (Acros Organics, Geel, Belgium) allowed quantitation of the capacity of HUVEC cells to generate NO (Ghebremariam et al. 2014; Jung et al. 2018). Cell images of DAF-FM, Hoechst 33342 and CellROX fluorescent dyes were analyzed with Fiji, the open-source processing program distribution of ImageJ (Schindelin et al. 2012). For DAF-FM and CellROX fluorescence, image intensity was averaged over the entire cell area.

***In Vivo Arterial Stiffness and Blood Pressure***

Aortic PWV was measured at baseline and following the intervention using Doppler ultrasonography (Indus Instruments, Webster, TX) under anesthesia (2% isoflurane with oxygen adjusted to maintain heart rate between 400-500 beats per minute), as previously described (Fleenor et al. 2012; Fleenor 2013; Gioscia-Ryan et al. 2018). PWV was calculated as the physical distance between probes divided by the difference in pre-ejections times (time between ECG R-wave and foot of the Doppler signal) of the thoracic and abdominal aortic regions.

Blood pressure was assessed *in vivo* at baseline and following the intervention using a CODA non-invasive tail-cuff system (Kent Scientific, Torrington, CT), as previously described (Fleenor et al. 2012).

***Motor function***

Motor function was assessed *in vivo* at baseline and following the intervention over 2 days, as previously described (Justice et al. 2013). On day 1, grip strength (normalized to body weight) was measured first as the average force over 5-10 trials (30 sec between trials) recorded at forepaw release from a custom grip strength device that included a force transducer (0.5 kg; Imada PS Series, Northbrook, IL) attached to a trapeze grip bar 1.5 mm in diameter. Briefly, each mouse was grasped by its tail, suspended just above the trapeze bar, and lowered until it successfully grasped the bar with both forepaws. A gradual horizontal tug was then applied until the mouse released its grip.

Later in the same day, mice performed 3 trials (≥60 min between trials) on an accelerating rota-rod test to determine maximal running speed. Briefly, mice were placed on a five-station accelerating rota-rod (Ugo Basile, Comerio, Italy), which was gradually accelerated from 4 to 40 rpm over 5 min. Latency to fall was recorded and maximal running speed was considered the speed achieved at the time of fall.

On day 2, mice performed 1 trial of an endurance rota-rod test. Mice with similar maximal running speeds (from the accelerating rota-rod test) were run at the same time on the five-station rota-rod, with speeds determined from the average maximal running speed of each set of five mice. Following a warm-up period (2 min at 25% max speed and 5 min at 50% max speed), mice ran at 75% of max running speed until fall or for up to 10 min (endurance period 1). If still running, the speed was then increased to 100% of max running speed until fall or for up to 20 min (endurance period 2). The latency to fall (i.e., time to fatigue) was determined from the start of endurance period 1.

***Insulin and Glucose Tolerance Tests***

Insulin sensitivity (insulin tolerance test) and glucose tolerance (glucose tolerance test) were assessed on two separate days following the intervention. Mice were fasted for 4 h prior to the insulin tolerance test (i.e., food was removed from their cages from 8 am to 12 pm) and for 6 h prior to the glucose tolerance test (i.e., food was removed from 8 am to 2 pm). For both tests, blood glucose was measured from the tail vein using a handheld glucometer (Accu-Chek® Nano Meter; Roche Diabetes Care, Inc., Indianapolis, IN) immediately prior to the tests and at 20-30 min intervals for up to 2 h following IP injection of insulin (0.75 U insulin/kg body weight; NovoLog®FlexPen®, Novo Nordisk, Inc., Princeton, NJ) or glucose (2 g D-glucose/kg body weight; Sigma-Aldrich, Corp.).

***Western Immunoblotting***

Protein expression was determined in thoracic aorta and quadriceps muscle lysates using either traditional Western immunoblotting [to detect nitrotyrosine (NT), Cu/Zn superoxide dismutase (SOD1), manganese superoxide dismutase (SOD2), phosphorylated and total p65 subunit of NF-κB, phosphorylated and total 5’ AMPK, and GAPDH loading control] or capillary electrophoresis Western detection (to detect the p67 subunit of NADPH oxidative and ERK loading control). For traditional immunoblotting, 20 μg of protein were loaded onto SDS gels, separated by electrophoresis, and transferred onto nitrocellulose membranes. Membranes were incubated in blocking buffer (TBS-T with 5% w/v dry milk powder) for ≥1 h, incubated overnight at 4°C in primary antibody, washed 3 times with TBS-T, incubated with the appropriate HRP-conjugated secondary antibody, then washed three times in TBS-T. Target proteins were then detected by chemiluminescent imaging after incubation in ECL Western Blotting Substrate (ThermoFisher Scientific, Inc., Waltham, MA). Primary antibodies for traditional Western blotting aortic protein targets were anti-NT (1:500; Abcam, Cambridge, UK; cat# ab7048), anti-SOD1 (1:1000; Enzo Life Sciences, Inc., Ann Arbor, MI; cat# ADI-SOD-101-E), anti-SOD2 (1:1000, Enzo Life Sciences, Inc.; cat# ADI-SOD-111-D), p65 subunit of p-NF-κB [1:1000; Cell Signaling Technology (CST), Beverly, MA; cat# 7F1], total p65 subunit of NF-κB (1:1000; CST; cat# C22B4), anti-p-AMPK (1:1000; CST; cat# T172), anti-AMPK (1:1000; CST; cat# 23A3), and anti-GAPDH (1:1000; CST; cat# 14C10). For quadriceps muscle targets, primary antibodies used were anti-p-AMPK (1:1000; CST; cat# T172), anti-AMPK (1:1000; CST; cat# 23A3), anti-NT (1:500; Abcam; cat# ab7048), anti-SOD1 (1:1000; Enzo Life Sciences, Inc.; cat# ADI-SOD-101-E), anti-SOD2 [1:1000; Enzo Life Sciences, Inc.; cat# ADI-SOD-111-D) and anti-GAPDH [1:1000; CST; cat# 14C10). For all Western targets, relative intensity was normalized against intensity of GAPDH signal for each sample.

Capillary electrophoresis Western detection was measured with use of a Wes instrument (ProteinSimple, San Jose, CA). Aliquots of aortic cell extract containing 2.5 µg of protein was separated by capillary electrophoresis. Electrophoretic separation and immunodetection were performed automatically using the default settings, except primary antibody incubation phase was extended to 150 minutes. Samples, blocking reagent, wash buffer, primary antibodies, secondary antibodies, and chemiluminescent substrate were aliquoted by the instrument. Resulting data was analyzed with the installed Compass software (ProteinSimple). Primary antibodies used to probe targets in aorta lysates by capillary electrophoresis include anti-p67 subunit of NADPH oxidase [1:100; Abcam; cat# ab109366] and anti-ERK 1 [1:1; ProteinSimple; cat# 042-486]. For p67, relative intensity was normalized against intensity of ERK 1 signal in lysates. Electropherograms in figures are represented as pseudo blots generated using Compass software.

***Skeletal Muscle ADP:ATP Ratio***

ADP:ATP nucleotide ratio was measured using the bioluminescent ADP:ATP Ratio Assay Kit (Abcam, San Francisco, CA). 20 mg of quadriceps muscle tissue was washed twice in PBS, pH 7.4. In order to obtain a suspension of single cells, muscle samples were then incubated in PBS, pH 7.4 with 10 mM glucose, 10% FBS and 2 mg/mL type I collagenase (Sigma-Aldrich, Corp.) at 37°C for 60 min in 6 well culture plates. After incubation, digestion solutions were drawn through a pipette tip >10 times to ensure cell dispersion. Nucleotide ratio was then assessed in muscle cell suspensions according to kit protocol.

***Aorta and Skeletal Muscle Inflammatory Cytokines***

Concentrations of inflammatory cytokines were measured using commercially-available single-target (IL-6, IL-1ra and KC: R&D Systems, Minneapolis, MN) or multiplex ELISA kits (TNFα, IFNγ and IL-1β: Ciraplex® Mouse Cytokine Array 1, Aushon BioSystems, Inc., Billerica, MA) using 10-20 μg of thoracic aorta or quadriceps muscle lysate.

***Basal Pancreatic Insulin Content***

Insulin concentrations were measured in pancreas lysates using a commercially-available ELISA kit (Cusabio Technology, Houston, TX).

***Plasma Metabolomics***

**Sample Preparation & UHPLC-MS – hydrophilic metabolite analysis.** Prior to LC-MS analysis, samples were placed on ice and a 20 µL aliquot was diluted with 480 µL of ice-cold methanol/acetonitrile/water (5/3/2). Extractions were performed and resulting samples analyzed using a 5 min C18 gradient on a Thermo Vanquish-Q Exactive system (San Jose, CA, USA), as previously described (Gehrke et al. 2019).

**Sample Preparation – lipid analysis.** Prior to LC-MS analysis, samples were placed on ice and a 10 uL aliquot was diluted with 90 uL of ice-cold methanol. Suspensions were vortexed to mix and placed at -20°C for 30 minutes. Insoluble material was removed by centrifugation at 12,000 g for 10 min at 4°C. Supernatants were isolated and diluted 1:1 (v/v) with 10 mM ammonium acetate for analysis by UHPLC-MS (D'Alessandro et al. 2019).

**UHPLC-MS lipid analysis.** Samples were analyzed on a Thermo Vanquish UHPLC system (San Jose, CA, USA) coupled online to a Thermo Q Exactive mass spectrometer (Bremen, Germany). Lipids were resolved over a Waters ACQUITY HSS T3 column (2.1 x 150 mm, 1.8 µm) using an aqueous phase (A) of 25% acetonitrile and 5 mM ammonium acetate and a mobile phase (B) of 50% isopropanol, 45% acetonitrile and 5 mM ammonium acetate. Samples were eluted from the column using either the solvent gradient: 0-1 min 25% B and 0.3 mL/min; 1-2 min 25-50% B and 0.3 mL/min, 2-8 min 50-90% B and 0.3 mL/min, 8-10 min 90-99% B and 0.3 mL/min, 10-14 min hold at 99% B and 0.3 mL/min, 14-14.1 min 99-25% B and 0.3 mL/min, 14.1-16.9 min hold at 25% B and 0.4 mL/min, 16.9-17 min hold at 25% B and resume flow of 0.3 mL/min. The mass spectrometer was operated in negative ion mode, scanning in Full MS mode (2 μscans) from 150 to 1500 m/z at 70,000 resolution, with 4 kV spray voltage, 45 shealth gas, 15 auxiliary gas. Samples were analyzed in randomized order with a technical mixture injected incrementally to qualify instrument performance. Acquired data was then converted from .raw to .mzXML file format using RawConverter. Metabolites were assignments using Maven (Princeton, NJ, USA) (Nemkov et al. 2017; Clasquin et al. 2012)**.**

***Statistical Analyses***

Statistical analyses were performed in Prism 7 (GraphPad Software, Inc., LaJolla, CA). Data were first assessed for normality (Shapiro-Wilk normality test, p>0.05) within groups. Differences in carotid artery dose responses, PWV, blood pressure (SBP and DBP), motor function (time to fatigue and grip strength), and metabolic function (blood glucose responses to insulin and glucose tolerance tests) were assessed using two-way mixed design analysis of variance (ANOVA) with a between factor of group and repeated factor of either dose (carotid artery EDD and EID), time into the intervention (i.e., pre vs. post; PWV, BPs, motor function), or time into the test (blood glucose responses). When significant interactions were detected, pairwise comparisons were made using the Holm-Sidak post-hoc test. Differences across animal groups in morphological characteristics, plasma IL-37 concentrations, peak EDD, NO-mediated dilation, HUVEC serum exposure NO bioavailability and ROS production, Western blot markers, ADP/ATP ratio, inflammatory cytokines, and plasma metabolites were assessed using Student’s unpaired t-test. Significance was set to α=0.05. Unless otherwise noted, data are presented as mean ± S.E.M.

**References**

Clasquin MF, Melamud E & Rabinowitz JD (2012) LC-MS data processing with MAVEN: a metabolomic analysis and visualization engine. *Curr Protoc Bioinformatics* Chapter 14, Unit14.11–14.11.23.

D'Alessandro A, Reisz JA, Zhang Y, Gehrke S, Alexander K, Kanias T, Triulzi DJ, Donadee C, Barge S, Badlam J, Jain S, Risbano MG & Gladwin MT (2019) Effects of aged stored autologous red blood cells on human plasma metabolome. *Blood Adv* 3, 884–896.

Fleenor BS (2013) Large elastic artery stiffness with aging: novel translational mechanisms and interventions. *Aging Dis* 4, 76–83.

Fleenor BS, Sindler AL, Eng JS, Nair DP, Dodson RB & Seals DR (2012) Sodium nitrite de-stiffening of large elastic arteries with aging: role of normalization of advanced glycation end-products. *Exp Gerontol* 47, 588–594.

Gehrke S, Rice S, Stefanoni D, Wilkerson RB, Nemkov T, Reisz JA, Hansen KC, Lucas A, Cabrales P, Drew K & D'Alessandro A (2019) Red Blood Cell Metabolic Responses to Torpor and Arousal in the Hibernator Arctic Ground Squirrel. *J. Proteome Res.* 18, 1827–1841.

Ghebremariam YT, Huang NF, Kambhampati S, Volz KS, Joshi GG, Anslyn EV & Cooke JP (2014) Characterization of a fluorescent probe for imaging nitric oxide. *J Vasc Res* 51, 68–79.

Gioscia-Ryan RA, Battson ML, Cuevas LM, Eng JS, Murphy MP & Seals DR (2018) Mitochondria-targeted antioxidant therapy with MitoQ ameliorates aortic stiffening in old mice. *J Appl Physiol* 124, 1194–1202.

Jung IH, Kim SE, Lee Y-G, Kim DH, Kim H, Kim G-S, Baek N-I & Lee DY (2018) Antihypertensive Effect of Ethanolic Extract from Acanthopanax sessiliflorus Fruits and Quality Control of Active Compounds. *Oxid Med Cell Longev* 2018, 5158243–14.

Justice JN, Carter CS, Beck HJ, Gioscia-Ryan RA, McQueen M, Enoka RM & Seals DR (2013) Battery of behavioral tests in mice that models age-associated changes in human motor function. *Age (Dordr)* 36, 583–595.

Nemkov T, Hansen KC & D'Alessandro A (2017) A three-minute method for high-throughput quantitative metabolomics and quantitative tracing experiments of central carbon and nitrogen pathways. *Rapid Commun. Mass Spectrom.* 31, 663–673.

Schindelin J, Arganda-Carreras I, Frise E, Kaynig V, Longair M, Pietzsch T, Preibisch S, Rueden C, Saalfeld S, Schmid B, Tinevez J-Y, White DJ, Hartenstein V, Eliceiri K, Tomancak P & Cardona A (2012) Fiji: an open-source platform for biological-image analysis. *Nat Methods* 9, 676–682.
